# Calcium Sensing Receptor-Related Pathway Contributes to Cardiac Injury and the Mechanism of Astragaloside IV on Cardioprotection

**DOI:** 10.3389/fphar.2018.01163

**Published:** 2018-10-11

**Authors:** Meili Lu, Bin Leng, Xin He, Zhen Zhang, Hongxin Wang, Futian Tang

**Affiliations:** ^1^Key Laboratory of Cardiovascular and Cerebrovascular Drug Research of Liaoning Province, Jinzhou Medical University, Jinzhou, China; ^2^Internal Medicine-Cardiovascular Department, The First Affiliated Hospital of Jinzhou Medical University, Jinzhou, China

**Keywords:** astragaloside IV, calcium sensing receptor, cardiac hypertrophy, apoptosis, calcineurin

## Abstract

Activation of calcium sensing receptor (CaSR) contributes to cardiac injury, but the underlying mechanism has not yet been examined. Astragaloside IV (AsIV) was previously reported to exhibit protective effects against various myocardial injuries. The aim of the present study was to investigate the underlying mechanism of CaSR in cardiac hypertrophy and apoptosis and to evaluate whether the protective effect of AsIV against myocardial injury is associated with CaSR and its related signaling pathway. *In vivo* and *in vitro* myocardial injury was induced by isoproterenol (Iso) or GdCl_3_ (a CaSR agonist) in rats and heart H9C2 cells. Cardiac cell hypertrophy, apoptosis, function, Mitochondrial Membrane Potential (MMP), mitochondrial ultrastructure, and [Ca^2+^]_i_, as well as the protein expression of CaSR, calcium/calmodulin-dependent protein kinase II (CaMKII), calcineurin (CaN), sarcoplasmic reticulum Ca^2+^-ATPase2a (SERCA2a), and the inositol 1,4,5-trisphosphate receptor (IP3R), were measured *in vivo* and/or *in vitro*. The results showed that AsIV attenuated cardiac hypertrophy and apoptosis and attenuated impairments in cardiac function, mitochondrial structure, and MMP induced by Iso or GdCl_3_ in rat myocardial tissue and H9C2 cells. Importantly, AsIV treatment inhibited the enhancement of [Ca^2+^]_i_ and CaSR expression induced by Iso or GdCl_3_, an effect similar to that of the CaSR antagonist NPS2143. In addition, AsIV treatment repressed CaSR, CaMKII, and CaN activation and inhibited NFAT-3 nuclear translocation. Mechanistic analysis using lentivirus infection showed that CaSR overexpression activated the CaMKII and CaN signaling pathways and that this response was enhanced by Iso. The results suggested that CaSR-mediated changes in [Ca^2+^]_i_ and CaMKII and CaN signaling pathways contribute to cardiac hypertrophy and apoptosis and are involved in the protective effect of astragaloside IV against cardiac injury.

## Introduction

Heart failure is the end stage of many cardiovascular diseases and is a major public health problem. Cardiomyocyte apoptosis and hypertrophy contribute to the progression of heart failure. During apoptosis, the loss of hypertrophic cardiomyocytes exacerbates the myocardial contractility, aggravates myocardial fibrosis, reduces myocardial contractility, and ultimately contributes to heart failure (Li et al., 2009; Xu et al., 2011). Hypertrophic cardiomyocyte impairs the coordination of myocardial contraction and predisposes individuals to heart failure and sudden death. Hypertrophy of cardiomyocytes induced by β-adrenergic receptor stimulation accelerates the progress from compensated hypertrophy to heart failure, and the mechanisms are related to calcium homeostasis and to complex interactions in intracellular signaling pathways (Dewenter et al., 2017; Khalilimeybodi et al., 2017; Kim et al., 2017).

Calcium sensing receptor (CaSR) is a member of the G Protein-Coupled Receptors (GPCRs) superfamily, that was found to be expressed in the hearts and neonatal rat cardiomyocyte (Sun et al., 2006; Tfelt-Hansen et al., 2006) and is involved in systemic homeostasis of calcium and metal ions. Moreover, the CaSR in the cardiovascular system plays an important role in pulmonary hypertension, atherosclerosis, and ischemia/reperfusion injury (Li et al., 2012; Smith et al., 2016). Previous studies have shown that CaSR is involved in myocardial ischemia reperfusion injury through MAPK activation, phospho-PKCδ translocation to the mitochondria, calcium overload, caspase-3 activity, and Fas death receptor pathways (Jiang et al., 2008; Lu et al., 2010; Zheng et al., 2011). Subsequent studies have revealed the expression patterns and the critical role of CaSR in cardiac hypertrophy and apoptosis and have demonstrated that CaSR induces myocardial apoptosis through a mitochondrial dynamics-mediated apoptotic pathway in hypertensive hearts, aggravates myocardial hypertrophy through activation of autophagy, and induces Ca^2+^ release from the sarcoplasmic reticulum into the mitochondria in a rat model of heart failure (Lu et al., 2013; Hong et al., 2017). Although some progress has been made in understanding the role of CaSR in the cardiovascular system, the exact mechanism by which CaSR participates in cardiac hypertrophy and the downstream signaling pathway is not fully understood.

Astragaloside IV (AsIV), a purified small molecular saponin, is one of the major and active components in *Astragalus membranaceus.* Several studies have indicated that it has a wide spectrum of pharmacological effects including anti-inflammatory effects, antioxidative effects, regulatory effects on energy metabolism, and anti-apoptotic effects (Guo et al., 2016; Jiang et al., 2017; Li et al., 2017, 2018). These beneficial pharmacological and biochemical activities confer AsIV a potential therapeutic role in cardiovascular diseases. For instance, AsIV could improve cardiac function, alleviate ventricular remodeling via stimulating fatty acid β-oxidation, improve mitochondrial function and regulate Ca^2+^ homeostasis in rat model of heart failure (Tang et al., 2018). In addition, AsIV has been report to reduce the severity of myocarditis, attenuate cardiac inflammation, attenuate cardiac fibrosis induced by coxsackievirus B3 (CVB3), an effect that was mediated by inhibition of nuclear factor-kappaB (NF-κB) and transforming growth factor-β1(TGF-β1) signaling (Gui et al., 2015). Consistent with the results of these studies, we previously demonstrated a cardioprotective role of AsIV in Iso-induced cardiac hypertrophy that was at least partly attributed to the inhibition of calpain-1 activation and the TLR4/NF-κB signaling pathway (Yang et al., 2013; Mei et al., 2015). However, the effect of AsIV on Ca^2+^ homeostasis and CaSR in Iso-induced apoptosis and hypertrophy remains poorly understood. Therefore, we conducted the present study to test whether CaSR contributes to Ca^2+^ homeostasis and the Ca^2+^-mediated hypertrophic and apoptotic signaling pathway and whether this molecular mechanism is involved in the protective effect of AsIV against Iso induced myocardial injury.

## Materials and Methods

### Materials

AsIV was obtained from Nanjin Jingzhu Biotechnology Company (purity >98% measured by HPLC; Nanjing, China). Iso(I5627), GdCl_3_(G7532),and 2-APB(D9754), Dimethyl Sulfoxide (DMSO) and rhodamine-labeled phalloidin were purchased from Sigma-Aldrich (St. Louis, MO, United States). NPS2143 (S2633) was purchased from Selleck Chemicals (Houston, TX, United States). Antibodies against CaMKII(AB11287), SERCA2a (AB41825), and IP3R(AB12382) were purchased from Absci (Baltimore, MD, United States). Antibodies against NFAT-3 (ab99431), GATA-4 (ab84593), Bcl-2 (ab196495), and Bax(ab5313) were purchased from Abcam (Cambridge, MA, United States). Antibodies against CaN (13198-2-AP), CaSR (19125-1-AP), and β-actin (60008-1-Ig) were purchased from Proteintech Biotechnology (Wuhan, China). Nuclear and mitochondrial extraction kits were purchased from Vazyme Biotech Co., Ltd. (Nanjing, China). A 5,5′,6,6′-tetrachloro-1,1′,3,3′-tetraethyl-benzimidazol-carbocyanine iodide (JC-1) kit and Fluo-4 were obtained from the Beyotime Institute of Biotechnology (Nanjing, China). A terminal deoxynu-cleotidyl Transferase-Mediated dUTP Nick-End Labeling (TUNEL) kit was purchased from Roche (Darmstadt, Germany). TRIzol reagent, ANP and BNP primers were obtained from TaKaRa Biotechnology Co. (Dalian, China).

### Animal Experiments

The experimental protocols were approved by the Committee of Jinzhou Medical University for the Use of Experimental Animals for Research and Teaching. 60 Male Sprague-Dawley rats, weighting 220 to 250 g, were purchased from the Animal Center of Jinzhou Medical University (Certificate No. SCXK 2017-0003). The rats were housed under a 12 h:12 h light:dark cycle at 22 ± 2°C with 65–69% humidity and received a standard diet and water *ad libitum*. After 3 days preadaptation the rats were randomly divided into 6 groups (*n* = 10): (1) the Con group; (2) the Iso (10 mg/kg/d, i.p.) group; (3) the Iso + AsIV group; (4) the NPS2143 (1 mg/kg/d, i.p.) + Iso group; (5)the GdCl_3_ (10 mg/kg/d, i.p.) group; and (6) the GdCl_3_ + AsIV group. Rats in the AsIV group were gavaged with 80 mg/kg of AsIV suspended in 0.5% sodium carboxymethylcellulose (CMC) 1 day before Iso and GdCl_3_ administration, and all the drugs were given for 14 days. The dosages of GdCl_3_ and NPS2143 were selected according to previous studies (Zhao et al., 2011; Yamamura et al., 2012).

### Heart Weight Index Measurement

All animals were weighted and anesthetized with 20% urethane (0.5 ml/100 g, i.p.) at the end of the experiment. Then, the hearts of the rats were immediately harvested, rinsed in ice-cold 0.9% NaCl solution, dissected and weighed. The heart-weight index (the ratio of the heart weight to the body weight, HW/BW) and the left ventricle-weight index (the ratio of the left ventricular weight to the body weight, LVW/BW) were calculated separately. The heart tissues were weighed on a balance and then immediately put into liquid nitrogen or 4% formaldehyde for the next experiments.

### Electron Microscopy Analysis

Rats were perfused with 2.5% glutaraldehyde, and the heart tissues were taken, cut into <1 mm^3^ pieces, and fixed in 3% glutaraldehyde for 4 h and 1% osmic acid for 2 h. Then, the heart tissues were dehydrated with ethanol and propylene oxide, embedded in Epon-812, cut into ultrathin sections, stained with uranyl acetate and lead citrate and observed with a Philips CM 120 electron microscope (Amsterdam, Holland).

### HE and TUNEL Staining

Hearts tissues were fixed in 4% formaldehyde for 24 h and were then embedded in paraffin, cut into 5 μm sections, and stained with Hematoxylin-Eosin (HE). The TUNEL assay was performed according to the manufacturer’s protocol. The index of apoptosis was expressed as the number of positively stained apoptotic cardiomyocytes/the total number of cardiomyocytes counted × 100%.

### Echocardiography

Heart function was evaluated at the end of the experiment using a Siemens Acuson SC2000 high-frequency ultrasound system (Siemens, Inc., Berlin, Germany). The rats were anesthetized with inhaled isoflurane, and the LV Ejection Fraction (LVEF), LV fractional shortening (LVFS), and LV Internal Diastolic Diameter (LVIDd) were measured and analyzed using the M-mode.

### Cell Culture and Estimation of Cell Volume

H9C2 cells were obtained from Wuhan Boster Biotech Company (Wuhan, China) and propagated in Dulbecco’s Modified Eagle’s Medium (DMEM, Gibco) supplemented with 10% fetal bovine serum (FBS, HyClone) and 1% penicillin-streptomycin (Invitrogen). The cells were fixed with 4% paraformaldehyde at room temperature for 30 min, washed with PBS, and then treated with 0.5 μM rhodamine-labeled phalloidin for 30 min and 0.5 μM DAPI for 10 min. Then, the cells were examined and photographed using a fluorescence microscope.

### Lentiviral Overexpression of CaSR in H9C2 Cells

Rat H9C2 cells (5 × 10^4^/ml) were prepared and infected at a Multiplicity of Infection (MOI) of 50 with control or CaSR-overexpressing lentiviruses (Shanghai GeneChem) for 24 h at 37°C in the presence of 10 mg/ml polybrene. The H9C2 cells were then washed and cultured in fresh medium for 24 h for further analysis.

### ANP and BNP Measurement

Total RNA was extracted from H9C2 cells using TRIzol reagent according to the manufacturer’s protocol. The total RNA concentration was determined based on the absorption at 260 nm, and the purity was determined according to the A260/A280 ratios. The same amount of total RNA (2 μg) was used from each sample. RNA was reverse transcribed using AMV reverse transcriptase with random hexamers for 50 min at 42°C. The cDNA was denatured at 95°C for 5 s followed by 40 PCR cycles using ABI 7500 fast real time PCR system (Foster, CA, United States). The relative level of mRNA was calculated by the comparative C_T_ method with GAPDH mRNA as the invariant control.

### Apoptosis Assays

Apoptosis was detected using an annexin V-FITC apoptosis detection kit according to the manufacturer’s instructions. Briefly, H9C2 cells were seeded in a 25 cm^2^ flask and incubated with the indicated drugs for 24 h. The cells were harvested, washed twice with prechilled PBS and centrifuged at 2000 rpm for 5 min followed by staining with annexin V-FITC using an assay kit. The data were analyzed using the Bioconsort software (United States).

### Fluo-4/AM Measurements of Intracellular [Ca^2+^]_i_

H9C2 cells were cultured in confocal culture dishes and then loaded with 5 μM Fluo-4/AM for 30 min at 37°C in the dark. The H9C2 cells were then washed twice with Ca^2+^-free PBS to remove the extracellular Fluo-4/AM and incubated further in DMEM. Changes in [Ca^2+^]_i_ were measured by the fluorescence intensity induced by Fluo-4 in H9C2 cells recorded for 5 min using Leica TCS SP5II laser confocal scanning microscopy (Wetzlar, Germany).

### Measurement of Mitochondrial Membrane Potential (MMP)

Mitochondrial Membrane Potential (MMP) was determined by JC-1 staining. After the different treatments, H9C2 cells were incubated for 20 min in the presence of 2 μM JC-1 at 37°C. Then, the cells were washed twice with JC-1 buffer solution. Thereafter, labeled cells were analyzed and quantified by Leica DMI3000B fluorescence microscope (Wetzlar, Germany).

### Immunofluorescence

H9C2 cells were fixed in 4% paraformaldehyde, permeabilized with 0.5% Triton X-100, and blocked with 5% BSA for 30 min at room temperature. The cells were then incubated with a CaSR antibody (1:100) overnight at 4°C. The next day, the cells were washed three times and incubated with fluorescein isothiocyanate (FITC)-conjugated goat anti-rabbit secondary antibody for 1 h at 37°C in the dark. After being washed with PBS, the cells were treated with 0.5 μM rhodamine-labeled phalloidin for 30 min and 0.5 μM DAPI for 10 min. Then, the cells were examined and photographed using Leica DMI3000B fluorescence microscope (Wetzlar, Germany).

### Preparation of Protein Extracts and Western Blot Analysis

Nuclear and mitochondrial proteins were extracted from heart tissues and H9C2 cells using a nuclear and mitochondrial extraction kit according to the manufacturer’s instructions. The protein concentration was determined by the BCA method. After boiling the samples for 5 min, the protein samples were fractionated by SDS-PAGE (10–12% polyacrylamide gels), transferred to Polyvinylidene Fluoride (PVDF) membranes (Millipore, Bedford, MA) and blocked with 1% BSA for 2 h. The membranes were incubated with primary antibodies for CaSR, CaMKII, CaN, NFAT-3, GATA4, Bcl-2, Bax, and β-actin at room temperature for 1.5 h. Detection was performed with enhanced chemiluminescence reagents. The results were analyzed with Quantity One software (Bio-Rad Laboratories, Hercules).

### Statistics

All data are expressed as the means ± SEM. SPSS 17.0 software (Chicago, IL, United States) was used to analyze all the data. Statistical analysis was performed using one-way ANOVA followed by Bonferroni’s test. *P* < 0.05 was considered statistically significant.

## Results

### AsIV Inhibited Cardiac Hypertrophy and Corrected Cardiac Dysfunction Induced by Iso or GdCl_3_

**FIGURE 1:**
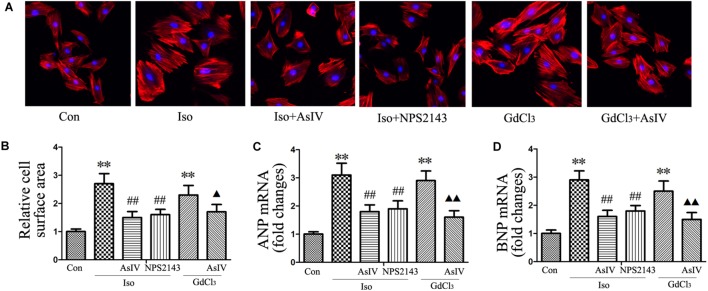
AsIV inhibited H9C2 cell hypertrophy induced by Iso or GdCl_3._**(A)** H9C2 cells were stained with rhodamine-labeled phalloidin and DAPI and then examined and photographed using a fluorescence microscope (magnification × 200). **(B)** Cell surface area was measured and analyzed with LAS Software (V4.3) (Leica, Germany). The bars represent the cellular size relative to that of the controls. **(C,D)** mRNA expression of ANP and BNP. The data are expressed as the means ± SEM, *n* = 4. ^∗∗^*P* < 0.01 vs. the Con group; ^##^*P* < 0.01 vs. the Iso group; and ^
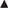
^*P* < 0.05, ^
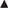

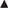
^*P* < 0.01 vs. the GdCl_3_ group.

**FIGURE 2:**
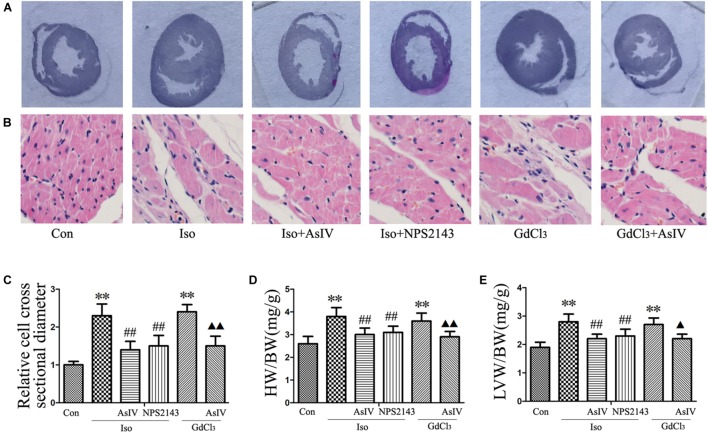
AsIV inhibited cardiac hypertrophy induced by Iso or GdCl_3_ in rats_._ **(A)** Left ventricular tissue section stained with H&E. **(B)** Representative example of H&E staining of heart tissue. **(C)** Statistical data on cell surface area shown by H&E staining and measured and analyzed with LAS Software. **(D,E)** Ratios of heart weight/body weight (HW/BW) and left ventricular weight/body weight (LVW/BW). Data are expressed as the means ± SEM, *n* = 4 for A–C; *n* = 8 for D and E. ^∗∗^*P* < 0.01 vs. the Con group; ^##^*P* < 0.01 vs. the Iso group; and ^
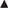
^*P* < 0.05, ^
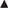

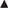
^*P* < 0.01 vs. the GdCl_3_ group.

To investigate the inhibitory effect of AsIV on cardiac hypertrophy, we created a hypertrophy model using Iso or GdCl_3_. The results showed that activation of CaSR with GdCl_3_ (30 μM) induced cardiac hypertrophy, as indicated by the increased cell surface area (**Figures 1A,B**) and increased mRNA expression of ANP and BNP in H9C2 cells (**Figures 1C,D**), effects that were similar to the effects of Iso (10 μM). Injecting rats with Iso or GdCl_3_ for 14 days induced cardiac hypertrophy, as indicated by the increased HW/BW and LVW/BW ratios (**Figures 2D,E**), the cardiomyocyte cross-sectional diameter and ventricular wall thickness (**Figures 2A–C**). The present study showed that 100 μM AsIV inhibited Iso-induced cardiac hypertrophy similarly to the CaSR antagonist NPS2143 (1 μM). In addition, the combination of AsIV with GdCl_3_ also ameliorated cardiac hypertrophy induced by GdCl_3_. Furthermore, cardiac function was evaluated individually by estimation of the LVEF, LVFS, and LVIDd through echocardiography. Compared with the parameters in the control group, rats injected with Iso or GdCl_3_ for 14 days resulted in decreased LVEF and LVFS and increased LVIDd, indicating hypertrophy in the decompensation phase, and cardiac function was impaired. However, the combination of AsIV with Iso or GdCl_3_ improved cardiac function, as evidenced by increased LVEF and LVFS and decreased LVIDd (**Table 1**).

**Table 1:** Parameters of cardiac function in rats.

Group	LVEF (%)	LVFS (%)	LVIDd (mm)
Con	77.45 ± 4.45	33.38 ± 3.84	6.18 ± 0.43
Iso	57.73 ± 5.44**^∗∗^**	20.10 ± 3.76**^∗∗^**	8.10 ± 0.36**^∗∗^**
Iso + AsIV	69.50 ± 5.63^##^	30.68 ± 4.37^##^	6.80 ± 0.34^##^
Iso + NPS2143	66.35 ± 5.65^#^	28.95 ± 2.38^##^	6.95 ± 0.39^##^
GdCl_3_	59.33 ± 7.01**^∗∗^**	21.45 ± 3.14**^∗∗^**	7.63 ± 0.57**^∗∗^**
GdCl_3_ + AsIV	70.67 ± 3.93^ 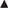 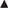 ^	30.55 ± 3.70^ 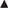 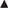 ^	6.92 ± 0.52^ 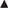 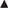 ^


*LVEF, left ventricular ejection fraction; LVFS, left ventricular fractional shortening; and LVIDd, left ventricular internal diastolic diameter. n = 4 rats for each group. ^∗∗^P < 0.01 vs. the Con group; ^##^P < 0.01, ^#^P < 0.05 vs. the Iso group; and.^
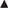

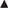
^P < 0.01 vs. the GdCl_*3*_ group.*

### AsIV Inhibited Apoptosis in Hypertrophied Heart Tissue and Cell

**FIGURE 3:**
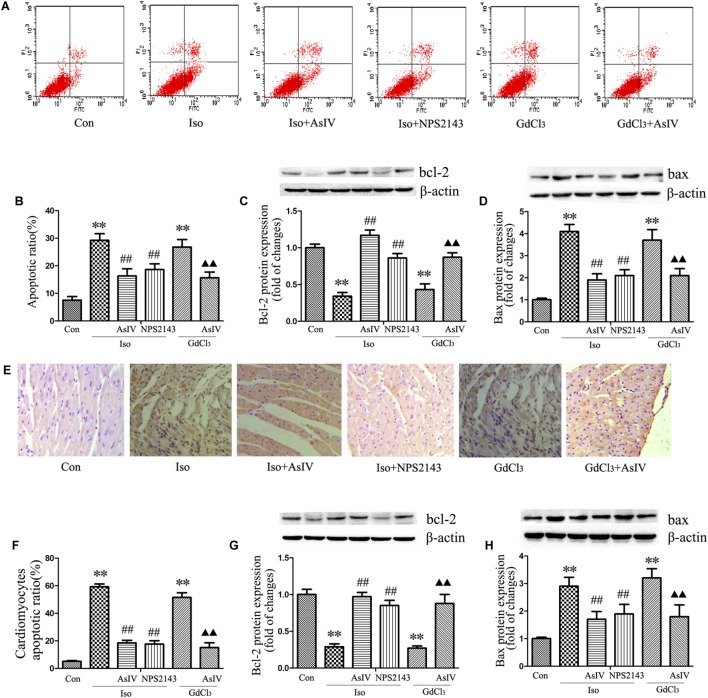
AsIV inhibited apoptosis in hypertrophied heart tissue and cells. **(A,B)** The level of apoptosis in H9C2 cells was examined using Annexin-V/PI staining and flow cytometry. The upper right area and the lower right area were regarded as apoptotic areas. **(C,D)** Bax and Bcl-2 protein expression assayed by western blot analysis in H9C2 cells. **(E,F)** Myocardial apoptosis assayed by TUNEL staining. TUNEL-positive cells were manifested as a marked appearance of dark brown apoptotic cell nuclei. **(G,H)** Bax and bcl-2 protein expressions assayed by western blot in myocardial tissue. The data are expressed as the means ± SEM, *n* = 4. ^∗∗^*P* < 0.01 vs. the Con group; ^##^*P* < 0.01 vs. the Iso group; and ^
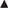
^*P* < 0.05, ^
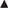

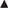
^*P* < 0.01 vs. the GdCl_3_ group.

Hypertrophic cardiomyocytes are more susceptible to apoptosis, promoting the transformation from hypertrophy to heart failure. To further explore the protective effect of AsIV against cardiac injury, we examined the apoptotic rate and the protein expression of Bax and Bcl-2. The results showed that AsIV decreased the number of Annexin-V/PI positive cells in the H9C2 cells (**Figures 3A,B**), reduced the number of TUNEL-positive cells in heart tissue (**Figures 3E,F**), upregulated Bcl-2 expression and downregulated Bax expression compared with these parameters in myocardial tissue (**Figures 3G,H**) and H9C2 cells (**Figures 3C,D**) treated with Iso or GdCl_3_ alone, and NPS2143 had anti-apoptotic effects similar to those of AsIV.

### AsIV Improved Mitochondrial Ultrastructure and MMP Dissipation

**FIGURE 4:**
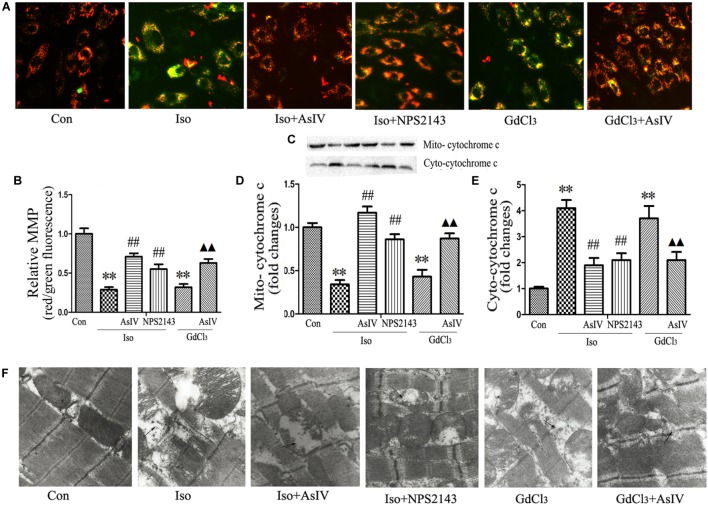
AsIV ameliorated mitochondrial dysfunction in heart tissue and H9C2 cells. **(A)** H9C2 cells were stained with JC-1, and mitochondrial membrane potential was assessed by fluorescence microscopy. **(B)** A decreased ratio of red fluorescence to green fluorescence indicates MMP dissipation. **(C–E)** Mitochondrial and cytoplasmic cytochrome c protein expression assayed by western blot in H9C2 cells. **(F)** EM was used to detect ultrastructural changes in the left ventricular tissue after the different treatments (magnification × 15,000). The arrows indicate the regions of mitochondrial swelling, cristae breakage, and membrane fusion. The data are expressed as the means ± SEM, *n* = 4. ^∗∗^*P* < 0.01 vs. the Con group; ^##^*P* < 0.01 vs. the Iso group; and ^
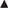
^*P* < 0.05, ^
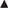

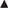
^*P* < 0.01 vs. the GdCl_3_ group.

Mitochondrial Membrane Potential (MMP) dissipation and cytochrome c release from the mitochondria to the cytoplasm prove the involvement of the mitochondrial apoptotic pathway in heart failure. The present study observed the effect of AsIV on Iso or GdCl_3_-induced MMP dissipation and cytochrome c release. In live cells, the mitochondria appear red due to the aggregation of accumulated JC-1, while in apoptotic and dead cells, the dye remains in its monomeric form, and the mitochondria appear green. Therefore, a decreased ratio of red fluorescence to green fluorescence indicates MMP dissipation. The results showed that Iso- or GdCl_3_-induced decreases in MMP (**Figures 4A,B**) and mitochondrial cytochrome c content (**Figures 4C–E**) were ameliorated by AsIV treatment. In addition, electron microscopy showed that pretreatment with AsIV ameliorated Iso- or GdCl_3_-induced mitochondrial lesions including mitochondria swelling, cristae breakage, and membrane fusion in cardiac tissue (**Figure 4F**).

### AsIV Decreased CaSR Expression in Heart Tissue and Cells

**FIGURE 5:**
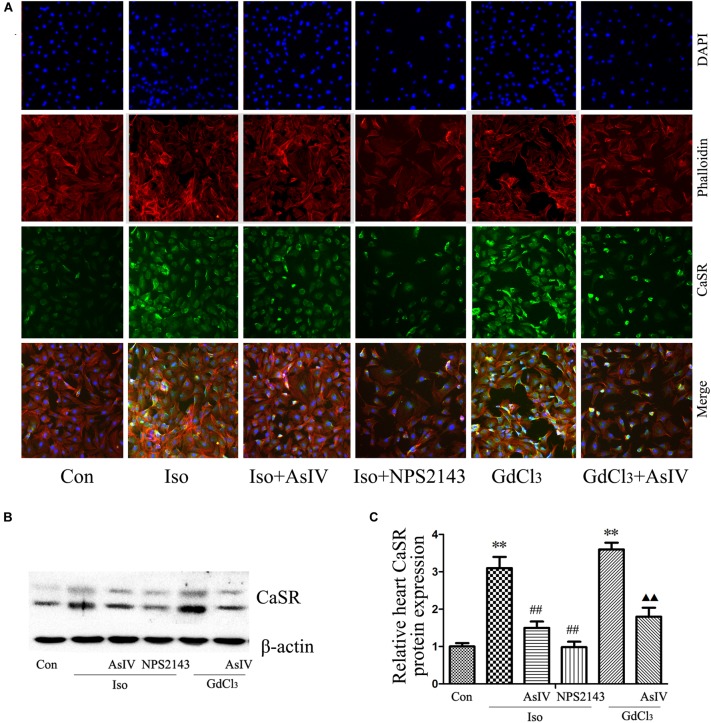
AsIV decreased CaSR expression in heart tissue and cells. **(A)** The H9C2 cells were incubated with a CaSR antibody overnight at 4°C, incubated with fluorescein isothiocyanate (FITC)-conjugated goat anti-rabbit secondary antibody, and then treated with rhodamine-labeled phalloidin and DAPI. The cells were examined and photographed using a fluorescence microscope. **(B,C)** CaSR protein expression as assayed by western blot in myocardial tissue. The data are expressed as the means ± SEM, *n* = 4. ^∗∗^*P* < 0.01 vs. the Con group; ^##^*P* < 0.01 vs. the Iso group; and ^
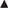
^*P* < 0.05, ^
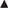

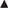
^*P* < 0.01 vs. the GdCl_3_ group.

Calcium sensing receptor (CaSR) activation contributes to the cardiac injury induced by Iso. To further explore the mechanism underlying the attenuation of cardiac injury by AsIV, the present study investigated the effect of AsIV on CaSR expression in heart tissue and H9C2 cell. The results of immunofluorescence analysis in H9C2 cells (**Figure 5A**) and western blot analysis in myocardial tissue (**Figures 5B,C**) showed that the combination of Iso with AsIV significantly suppressed CaSR expression compared with the expression after Iso alone, an effect similar to that of NPS2143. In addition, AsIV suppressed the CaSR expression induced by GdCl_3_. The results suggested that AsIV exerted a protective effect via inhibiting CaSR activation.

### AsIV Regulated [Ca^2+^]_i_ Induced by Iso and GdCl_3_

**FIGURE 6:**
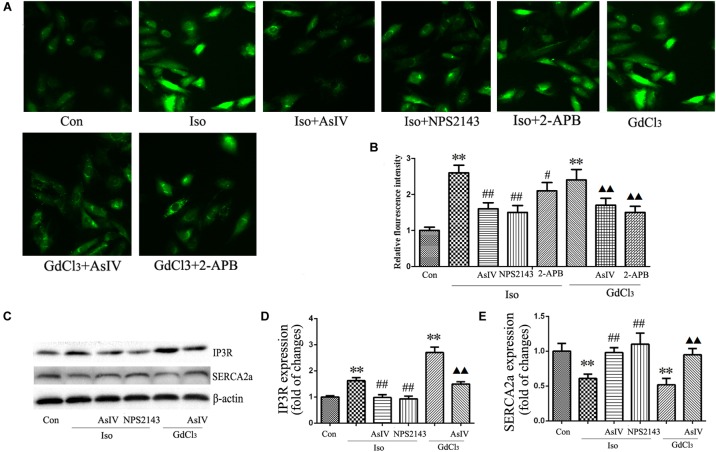
AsIV regulated [Ca^2+^]_i_ induced by Iso and GdCl_3_. **(A)** Changes in the intensity of fluorescence of [Ca^2+^]_i_ were recorded with a laser scanning confocal microscope under different treatment conditions. **(B)** The bars represent the fluorescence relative to that of the controls. **(C–E)** IP3R and SERCA2a protein expression assayed by western blot analysis in myocardial tissue. Data are expressed as mean ± SEM, *n* = 4. ^∗∗^*P* < 0.01 vs. Con group, ^##^*P* < 0.01 vs. Iso group, and ^
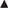
^*P* < 0.05, ^
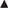

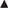
^*P* < 0.01 vs. GdCl_3_ group.

Activation of CaSR increases [Ca^2+^]_i_ through the PLC-IP3 pathway. To determine the effects of AsIV on increases in [Ca^2+^]_i_ induced by Iso and CaSR activation, the cells were stained with Fluo-4/AM, and 20 μM of 2-APB (an IP3R inhibitor) was used. The results showed that both Iso and GdCl_3_ increased [Ca^2+^]_i_, an effect that was inhibited by pretreatment with AsIV. Interestingly, 2-APB completely prevented the GdCl_3_-induced enhancement of [Ca^2+^]_i_, but moderately prevented the Iso-induced enhancement of [Ca^2+^]_i_, indicating that the increased [Ca^2+^]_i_ induced by Iso may be partly due to CaSR activation (**Figures 6A,B**).

The activity of the calcium pump SERCA2a is considerably reduced in the hypertrophic heart, and changes in SERCA2a activity during hypertrophy contributes substantially to Ca^2+^ overload. The present study showed the regulatory effect of AsIV on [Ca^2+^]_i_ and CaSR expression, so we investigated whether AsIV could potentially affect heart SERCA2a and IP3R activity in myocardial tissues. Compared with the expression in the control group, SERCA2a protein expression decreased and IP3R expression increased in the Iso and GdCl_3_ groups, and these changes were prevented by AsIV treatment (**Figures 6C–E**).

### AsIV Downregulated the CaMKII and CaN Pathways in Heart Tissue and Cells

**FIGURE 7:**
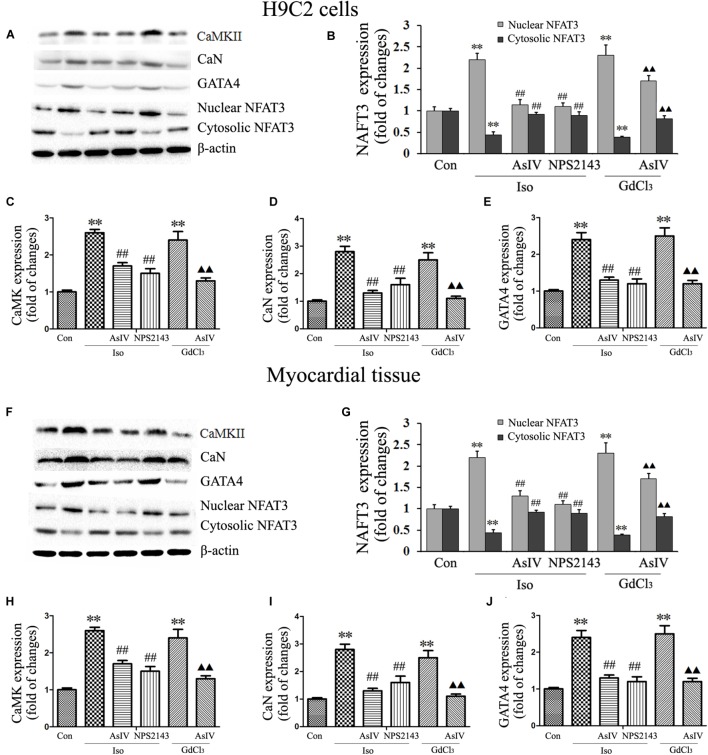
AsIV downregulated the CaMKII and CaN pathways in heart tissue and cells. **(A)** Representative western blots of CaMKII, CaN, GATA-4 and NFAT-3 in H9C2 cells. **(B–E)** Statistical data on the protein expression of CaMKII, CaN, GATA-4 and NFAT-3 in H9C2 cells. **(F)** Representative western blots of CaMKII, CaN, GATA4 and NFAT-3 in myocardial tissues. **(G–J)** Statistical data on the protein expression of CaMKII, CaN, GATA-4 and NFAT-3 in myocardial tissues. The data are expressed as the means ± SEM, *n* = 4. ^∗∗^*P* < 0.01 vs. the Con group; ^##^*P* < 0.01 vs. the Iso group; and ^
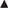
^*P* < 0.05, ^
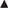

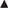
^*P* < 0.01 vs. the GdCl_3_ group.

Both hypertrophy and apoptosis are regulated by Ca^2+^ and by complex interactions in intracellular signaling pathways. In the present study, we determined the expression of CaMKII (**Figures 7A,C,F,H**), CaN (**Figures 7A,D,F,I**), and GATA-4 (**Figures 7A,E,F,J**) and the nuclear translocation of NFAT-3 (**Figures 7A,B,F,G**), all of which are implicated in the development of cardiac hypertrophy and apoptosis and are mediated by [Ca^2+^]_i_. Our results showed that pretreatment with AsIV significantly inhibited the Iso-induced activation of CaMKII, CaN, and GATA-4 and nuclear translocation of NFAT-3, an effect similar to that of NPS2143. Furthermore, CaSR activation with GdCl_3_ also activated CaMKII, CaN, and GATA-4 and promoted nuclear translocation of NFAT-3, effects that were inhibited by AsIV. These results suggested that the upregulation of CaSR induced by Iso increased [Ca^2+^]_i,_ and activated the CaMKII and CaN pathways, subsequently inducing cardiac apoptosis and hypertrophy. AsIV downregulated CaSR expression, inhibited increases in [Ca^2+^]_i_, inhibited the CaMKII and CaN pathways, and subsequently alleviated the cardiac injury induced by Iso.

### Regulatory Effect of CaSR on CaMKII and CaN Pathways Induced by Iso

**FIGURE 8:**
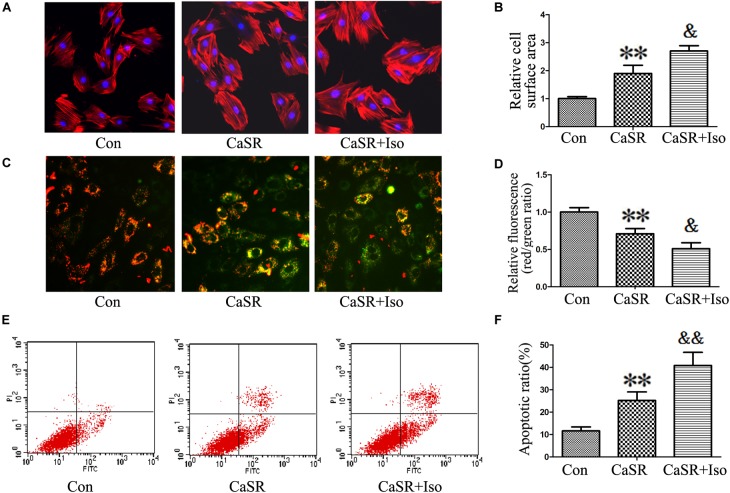
Regulatory effect of CaSR overexpression on cell hypertrophy and apoptosis. **(A,B)** H9C2 cells were stained with rhodamine-labeled phalloidin and DAPI, and the cell surface area was measured and analyzed with LAS Software (V4.3) (Leica, Germany). The bars represent the cell size relative to that of the controls. **(C,D)** H9C2 cells were stained with JC-1, and the bars represent the fluorescence relative to that of the controls. **(E,F)** The level of apoptosis in H9C2 cells was examined using Annexin-V/PI staining and flow cytometry. The data are expressed as the means ± SEM, *n* = 4. ^∗∗^*P* < 0.01 vs. the Con group; and ^&^*P* < 0.05; ^&&^*P* < 0.01 vs. the shCaSR group.

**FIGURE 9:**
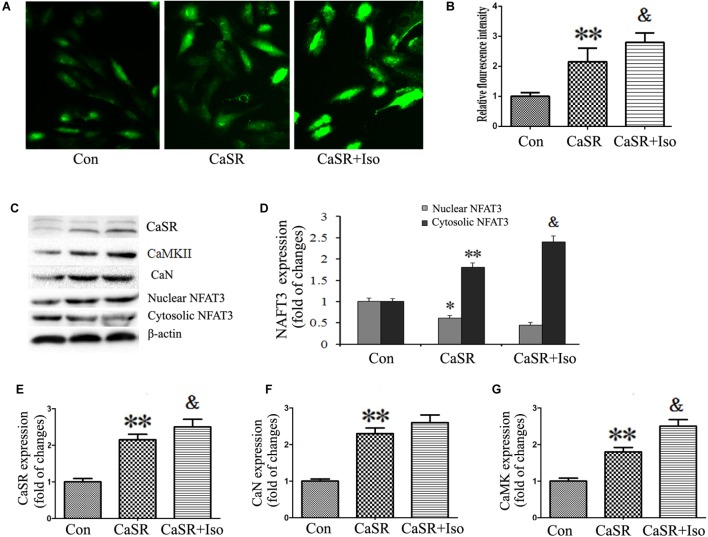
Regulatory effect of CaSR activation on [Ca^2+^]_i_ and the CaMKII and CaN pathways. **(A)** Changes in the intensity of fluorescence of [Ca^2+^]_i_ were recorded with a laser scanning confocal microscope under different treatment conditions. **(B)** The bars represent the fluorescence relative to that of the controls. **(C–G)** CaSR, CaMKII, CaN and NFAT-3 protein expression assayed by western blot in H9C2 cells. The data are expressed as the means ± SEM, *n* = 4. ^∗∗^*P* < 0.01 vs. the Con group; ^&^*P* < 0.05 vs. the CaSR group.

Finally, to confirm the regulatory effect of CaSR on the CaMKII and CaN pathways induced by Iso, we upregulated CaSR expression through lentivirus infection. The results showed that CaSR overexpression increased H9C2 cell size (**Figures 8A,B**), apoptotic ratio (**Figures 8E,F**), and [Ca^2+^]_i_ (**Figures 9A,B**), decreased H9C2 MMP (**Figures 8C,D**), and upregulated the CaMKII and CaN pathways (**Figures 9C–G**). Furthermore, the effects of CaSR activation on cardiac hypertrophy, apoptosis and the CaMKII and CaN pathways were enhanced by Iso administration. Combined with the abovementioned results, we demonstrated that the activation of Ca^2+^-dependent CaMKII and CaN pathways is involved in CaSR-induced cardiac hypertrophy and apoptosis.

## Discussion

**FIGURE 10:**
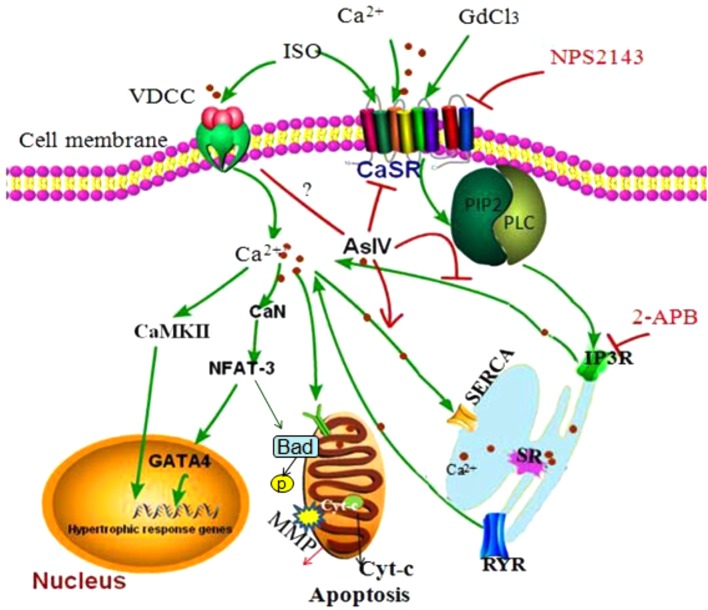
The diagram demonstrating the mechanism of cardioprotection by AsIV via downregulation of CaSR. The stimulation of cardiomyocytes by excessive extracellular Ca^2+^, Iso and GdCl_3_ induces overload of [Ca^2+^]_i_ through CaSR/IP3R pathway. The increased [Ca^2+^]_i_ leads to activation of CaMKII and CaN signaling pathway, resulting in cardiac hypertrophy and apoptosis. AsIV inhibits cardiac hypertrophy and apoptosis through downregulation of CaSR and IP3R expression and activation of SERCA 2a.

Apoptosis and hypertrophy of cardiomyocytes are the primary causes of heart failure and are known to be regulated by complex interactions in the intracellular signaling network. Through extensive computer simulations with various parameter distributions, it has been revealed that Ca^2+^, CaMKII, and NFAT are consistently found in all the perturbation analyses for cardiac hypertrophy and apoptosis (Liu Y.T. et al., 2016). Activation of CaSR has been reported to be involved in the development of various cardiovascular diseases including cardiac hypertrophy and apoptosis (Schepelmann et al., 2016; Paquot et al., 2017; Zhang et al., 2018), but the molecular mechanism of the involvement of CaSR activation in heart failure has not yet been clarified and thus needs to be further explored. In the present study, we investigated the role of CaSR activation in cardiac hypertrophy and apoptosis and the relationship between CaSR and Ca^2+^-dependent CaMKII and CaN signaling pathways both *in vivo* and *in vitro*. The results demonstrated that CaSR activation contributed to cardiac hypertrophy and apoptosis, and the effect of CaSR was mediated by the activation of Ca^2+^-dependent CaMKII and CaN signaling pathways. What is more, AsIV suppressed CaSR activation, downregulated Ca^2+^/CaMKII/CaN signaling pathways, and subsequently attenuated cardiac hypertrophy and apoptosis induced by Iso (**Figure 10**).

We previously reported that AsIV attenuated Iso-induced myocardial injury by inhibiting calpain-1 activation and the TLR4/NF-κB signaling pathway and by correcting energy biosynthesis dysfunction (Yang et al., 2013; Mei et al., 2015; Zhang et al., 2015). The present study confirmed the findings of our previous studies that AsIV treatment attenuates cardiac hypertrophy and apoptosis, as reflected by the (1) decreases in HW/BW, LVW/BW, cardiomyocyte cross-sectional diameter, ventricular wall thickness, and TUNEL-positive cells in myocardial tissue, and (2) decreases in cell size, ANP and BNP mRNA expression and Annexin-V/PI-positive cells in H9C2 cells. The novel finding is that the inhibitory action of AsIV was accompanied by downregulations in CaSR expression and [Ca^2+^]_i_, and the inhibitory effect of AsIV was similar to that of the CaSR antagonist NPS2143. It has been previously reported that CaSR activation with GdCl_3_ can induce cardiac hypertrophy and apoptosis (Lu et al., 2013). As an agonist of CaSR, GdCl_3_ gets involved in cardiac hypertrophy and apoptosis by activating CaSR. Activation of CaSR increases [Ca^2+^]_i_, then triggers activation of hypertrophic and apoptotic signaling pathways, which are regulated by [Ca^2+^]_i_. AsIV also attenuated GdCl_3_-induced cardiac hypertrophy and apoptosis through the downregulation of CaSR expression and [Ca^2+^]_i_. CaSR detects changes in extracellular calcium concentrations ([Ca^2+^]_o_). Increased levels of [Ca^2+^]_o_ induce the binding of Ca^2+^ to CaSR and activate the G-protein-PLC-IP 3 receptor pathway, which triggers the elevation of [Ca^2+^]_i_, thus initiating numerous effects (Brown and MacLeod, 2001). Under physiological conditions, Ca^2+^ enters cardiomyocytes through the L-type Ca^2+^ channel (LTCC) and the β-adrenergic receptor (β-AR), causing the release of a large amount of Ca^2+^ from the Sarcoplasmic Reticulum (SR) through activation of the RyR and leading to contraction. However, under some pathological conditions, such as pulmonary hypertension, myocardial ischemia, and cardiac hypertrophy, CaSR expression is upregulated, leading to [Ca^2+^]_i_ enhancement and thus aggravating the progression of these diseases (Guo et al., 2012, 2017; Zhang et al., 2016). Upon review of the previous studies, it was found that CaSR contributes to cardiac hypertrophy induced by angiotensin II, Iso, and transverse aortic constriction, and the mechanism may involve [Ca^2+^]_i_, the sarcoplasmic reticulum (ER), the mitochondrial death pathway and autophagy (Wang et al., 2008; Lu et al., 2013; Liu L. et al., 2016). Downregulation of CaSR with Calhex-231 and NPS2143 reduces vascular reactivity via inhibition of voltage-gated Ca^2+^ channels (Greenberg et al., 2016). Consistent with the results of previous studies, the present study demonstrated that CaSR contributed to cardiac hypertrophy and apoptosis induced by Iso but that the mechanism may be related to mitochondrial dysfunction and Ca^2+^-dependent CaMKII and CaN pathways.

Mitochondrial dysfunction contributes to multiple types of damage, including cardiac hypertrophy and apoptosis. The mitochondrial release of cytochrome c was analyzed to prove the involvement of the mitochondrial apoptotic pathway in heart failure. In our experiment, Iso administration destroyed mitochondrial structure in rats and reduced the MMP and mitochondrial cytochrome c in H9C2 cells, indicating mitochondrial dysfunction. Pretreatment with AsIV attenuated cardiac hypertrophy and apoptosis, improved cardiac function, and downregulated CaSR expression while also improving mitochondrial structure and MMP and inhibiting cytochrome c release from the mitochondria. A previous study indicated that CaSR activation caused Ca^2+^ release from the SR into the mitochondria and induced cardiomyocyte apoptosis and that changes in SERCA2a activity during HF contribute substantially to Ca^2+^ overload and mitochondrial dysfunction (Dong et al., 2017). However, the present study investigated [Ca^2+^]_i_ using Fluo-4/AM and found that the IP3Rs inhibitor 2-APB completely prevented the GdCl_3_-induced enhancement of [Ca^2+^]_i_ but only moderately prevented the Iso-induced enhancement of [Ca^2+^]_i_. Pretreatment with AsIV inhibited the enhancement of [Ca^2+^]_i_ induced by Iso and GdCl_3_. In addition, SERCA2a protein expression decreased and IP3R expression increased in the Iso and GdCl_3_ groups, effects that were prevented by AsIV treatment. These results suggested that the increased [Ca^2+^]_i_ induced by Iso may be partly due to CaSR activation, and inhibition of [Ca^2+^]_i_ contributes to the protective effect of AsIV against Iso-induced cardiac injury.

Increasing evidence has demonstrated that the pathway of cell hypertrophy and apoptosis induced by CaSR/Ca^2+^ is one of the major contributors to cardiac injury. To investigate the underlying mechanism, we evaluated the CaMKII and CaN signaling pathways, both of which were regulated by [Ca^2+^]_i_. CaMKII is involved in cardiac hypertrophy and apoptosis induced by Iso and testosterone (Bin-Dayel et al., 2016; Duran et al., 2017; Park et al., 2018), and at the epigenetic level, CaMKII inactivates the negative regulator of adverse cardiac remodeling histone deacetylase 4 (HDAC4), leading to transcriptional activation of the myocyte enhancer factor 2 (MEF2), and phosphorylates histone H3 (Backs et al., 2009; Awad et al., 2013). CaN triggers apoptosis and hypertrophy either by dephosphorylating NFAT to activate the subsequent transcription of apoptosis and hypertrophy genes or by dephosphorylating Bad and thereby facilitating the binding of Bad to anti-apoptotic proteins (Lu et al., 2014; Lin et al., 2017). The current study showed that upregulation of CaSR was accompanied by increased CaMKII and CaN expression and NFAT-3 nuclear translocation induced by Iso or GdCl_3_. NPS2143, an inhibitor of CaSR, not only downregulated CaSR expression but also inhibited CaMKII and CaN expression and NFAT-3 nuclear translocation induced by Iso. Finally, in order to further elucidate the molecular mechanism by which CaSR contributes to cardiac injury induced by Iso, we established a gain-of-function mutation in CaSR by lentivirus infection. As we expected, CaSR activation by lentivirus infection resulted in CaSR upregulation, [Ca^2+^]_i_ overload, and CaMKII and CaN pathway activation as well as cardiomyocyte hypertrophy and apoptosis. In addition, all of the aforementioned effects were enhanced by Iso, suggesting that regulation of [Ca^2+^]_i_ and Ca^2+^-dependent CaMKII and CaN pathways is the molecular mechanism by which CaSR contributes to cardiac injury induced by Iso. Furthermore, pretreatment with AsIV had an effect similar to that of NPS2143 and inhibited the activation of the CaMKII and CaN pathways, indicating that the protective effect of AsIV against cardiac injury may be partly mediated via the inhibition of CaSR, [Ca^2+^]_i_ elevation and Ca^2+^-dependent pathways. However, the present study has limitation. Although H9C2 cell line is commonly used as *in vitro* model of cardiomyocyte biology, it is a tumor line from atrial rat tumors with the disadvantages of not beating and not postmitotic like usually adult cardiomyocytes. So we will investigate the cardioprotective effect of AsIV using adult cardiomyocytes in further study. This study is of clinical significance for providing the evidence that AsIV might be potentially used in the prevention and treatment of hypertrophic cardiomyopathy.

## Conclusion

The present study demonstrated, for the first time, that CaSR contributes to cardiac hypertrophy and apoptosis by increasing [Ca^2+^]_i_ and activating Ca^2+^-dependent CaMKII and CaN pathways. The cardioprotective effect of AsIV on Iso-induced cardiac injury may be partly mediated via the inhibition of CaSR, [Ca^2+^]_i_ elevation and, Ca^2+^-dependent pathways. This study provides a new perspective for developing AsIV as a therapeutic candidate for cardiovascular disease.

## Author Contributions

HW and ML conceived and designed the experiments. ML, BL, and XH performed the experiments. ML and ZZ analyzed the data. ML and FT wrote or modified the paper. All authors contributed to and approved the final draft of the manuscript.

## Conflict of Interest Statement

The authors declare that the research was conducted in the absence of any commercial or financial relationships that could be construed as a potential conflict of interest.
